# Factors associated with happiness and life satisfaction among workers

**DOI:** 10.1192/j.eurpsy.2023.649

**Published:** 2023-07-19

**Authors:** K.-S. Na, E. Kim, E. Kim

**Affiliations:** ^1^Department of Psychiatry, Gachon University, Incheon; ^2^Department of occupational and environmental medicine, Korea Workers’ Compensation & Welfare Service, Ansan-si, Korea, Republic Of

## Abstract

**Introduction:**

In modern society, mental health in the workplace is increasingly considered an im-portant issue and a major political agenda. Many studies have reported negative mental health risk factors or psychopathologies such as depression, anxiety, and suicidal inclination among workers. Accordingly, there are ongoing debates on the importance of establishing a system to screen and treat psychopathologies, such as the assessment of depression and anxiety. However, the absence of psychopathology or negative psychiatric factors does not guarantee good mental health. Mental health is a more comprehensive and complex concept. According to the World Health Organization, mental health is the state in which an individual can cope with routine stressors in life, work productively, and contribute to their organizations. Hence, it is needed to directly measure workers’ mental health in terms of happiness and life satisfaction.

**Objectives:**

To comprehensively investigate workers’ mental health, we explored factors associated with happiness and life satisfaction among workers using nationally representative data.

**Methods:**

We performed multiple regression analysis, with happiness and life satisfaction set as the outcome measures, and sociodemographic factors and work-related factors as the predictive variables.

**Results:**

A total of 7,797 participants (4,428 men [56.8%]) with a mean age of 46.58 years (SD = 13.50) were included in the analysis. Job satisfaction (β = 0.154, p < 0.001) and self-rated health (β = 0.175, p < 0.001) were the most strongly associated with happiness. Organizational commitment, region of work, average monthly income, education level, and number of guaranteed leaves were also strongly associated with happiness and life satisfaction. Life satisfaction had the highest adjusted R^2^ at 0.423. The adjusted R^2^ for happiness and the ladder approach were 0.283 and 0.213, respectively. The variance inflation factor was below 10, and residuals were below 0.1 for all predictor variables.

**Conclusions:**

Our results indicated that personal and work-related factors were associated with the happiness and life satisfaction of workers. Among work-related factors, subjective, intrinsic rewards such as job satisfaction and organizational commitment were more strongly associated than external rewards such as average monthly income or guaranteed vacations. These findings may be useful foundational data in devising policies and interventions to promote workers’ happiness and life satisfaction.

**Disclosure of Interest:**

None Declared

